# Recent Development of Prebiotic Research—Statement from an Expert Workshop

**DOI:** 10.3390/nu9121376

**Published:** 2017-12-20

**Authors:** Giorgio La Fata, Robert A. Rastall, Christophe Lacroix, Hermie J. M. Harmsen, M. Hasan Mohajeri, Peter Weber, Robert E. Steinert

**Affiliations:** 1 DSM Nutritional Products Ltd., R & D Human Nutrition and Health, P.O. Box 2676, CH-4002 Basel, Switzerland; hasan.mohajeri@dsm.com (M.H.M.); peter-weber@unity-mail.de (P.W.); robert.steinert@dsm.com (R.E.S.); 2 Department of Food and Nutritional Science, The University of Reading, Whiteknights Campus, Reading RG6 6AP, UK; r.a.rastall@reading.ac.uk; 3 Department of Health Sciences and Technology, Laboratory of Food Biotechnology, Institute of Food, Nutrition and Health, ETH Zurich, CH-8092 Zürich, Switzerland; christophe.lacroix@hest.ethz.ch; 4 Department of Medical Microbiology, University Medical Center Groningen, 9713 GZ Groningen, The Netherlands; h.j.m.harmsen@umcg.nl; 5 Department of Surgery, Division of Visceral and Transplantation Surgery, University Hospital Zürich, 8091 Zürich, Switzerland

**Keywords:** gut microbiota, healthy gut, prebiotics and health benefits

## Abstract

A dietary prebiotic is defined as ‘a substrate that is selectively utilized by host microorganisms conferring a health benefit’. Although this definition evolved concomitantly with the knowledge and technological developments that accrued in the last twenty years, what qualifies as prebiotic continues to be a matter of debate. In this statement, we report the outcome of a workshop where academic experts working in the field of prebiotic research met with scientists from industry. The workshop covered three main topics: (i) evolution of the prebiotic concept/definition; (ii) the gut modeling in vitro technology PolyFermS to study prebiotic effects; and (iii) the potential novel microbiome-modulating effects associated with vitamins. The future of prebiotic research is very promising. Indeed, the technological developments observed in recent years provide scientists with powerful tools to investigate the complex ecosystem of gut microbiota. Combining multiple in vitro approaches with in vivo studies is key to understanding the mechanisms of action of prebiotics consumption and their potential beneficial effects on the host.

## 1. Introduction

The human intestinal microbiota is composed of 10^13^–10^14^ microorganisms (mainly bacteria) whose collective genome is defined as the ‘gut microbiome’ [1,2]. It is involved in several biological processes such as nutrient utilization and energy storage [3,4], resistance against infections [5], maturation and modulation of the immune system functions [6], and support of the neuroendocrine functions [7] and, thus, is critical to overall health status of the host [8,9,10,11,12].

The microbiota–host interaction starts at birth and remains relatively stable during adulthood while its stability decreases in the elderly [2,13] when chronic and acute perturbations frequently appear which have been described by some as dysbiosis [9,14]. The latter occurs also in the context of prevalent disorders such as obesity, diabetes, and metabolic inflammation [15] although it is not clear at the present time if this is association or causation.

The concept that gut microbiota could be modulated to improve human health was proposed as early as the beginning of the twentieth century when Elie Metchnikoff reported that Bulgarian peasants lived longer lives because of their yogurt consumption [16]. The idea that certain dietary components could influence the populations of specific bacterial groups in the gut and that this could impact on host health was further developed in the 1960s by Tomotari Mitsuoka [17,18]. Mitsuoka was the first to link composition of diet with bacterial population and activities and health, laying the foundation for the formulation of the prebiotic concept in the mid-1990s [19]. Indeed, an increasing number of recent studies shows an association between human gut microbiota perturbations and pathological conditions such as inflammatory bowel disease (IBD) [20], irritable bowel syndrome (IBS) [21], obesity [22], and inflammation [11,14,23]. If these associations prove to be causative relationships, then this suggests that a targeted modulation of the gut microbiota may offer preventative and maybe even novel therapeutic approaches. A proof of principle is the recent developments of transplantation of entire fecal microbiota in the treatment of *Clostridium difficile* [24,25].

In addition, nutritional interventions that can contribute to the establishment or maintenance of a healthy gut microbiota, including the consumption of ‘prebiotics’ are gathering more attention. The pioneering concept, as introduced by Gibson and Roberfroid in the 1990s, emphasizes the importance of diet in the modulation of the gut microbiota and its relationships to human health. Since then, the definition has been discussed and refined several times to accommodate emerging knowledge. Although the main features have mostly been retained, some of the criteria that need to be fulfilled for a food ingredient to qualify as prebiotic are still a matter of debate.

Considering our increasing knowledge of the intestinal microbiota and its importance to human health, academic experts in the field of prebiotic research met with scientists from industry to discuss the progress of prebiotic research beyond the traditional concept and how these developments may stimulate the field. The workshop covered a wide range of topics including (1) the history and evolution of the prebiotic concept; (2) available in vitro models of colonic fermentation to study prebiotic effects; and (3) compounds with microbiome-modulating properties, such as vitamins, that may impact host health.

## 2. Results

### 2.1. Evolution of the Prebiotic Definition

Glenn Gibson and Marcel Roberfroid first defined a prebiotic as ‘A non-digestible food ingredient that beneficially affects the host by selectively stimulating the growth and/or activity of one or a limited number of bacteria in the colon, and thus improves host health’ [19]. At that time, the understanding of the microbial ecosystem in the gut was still rather limited. Enumeration of bacteria by ‘selective’ media was the norm and DNA-based microbiology was just being introduced. Commonly, investigators counted around four or five functional groups and the total bacterial count and, thus, in this context the idea of selectively stimulating ‘one or a limited number of bacteria’ as described in the definition made perfect sense.

Nowadays, there is a deeper understanding of the gut microbiome and sequencing studies continue to reveal the extent of the diversity of the ecosystem. These techniques, however, can also have their flaws, resulting sometimes in a rather skewed picture without providing absolute cell counts. They can also underrepresent functionally significant bacterial groups such as bifidobacteria depending on methodological details [26]. In contrast, fluorescent probe-based (fluorescence in-situ hybridization (FISH) enumeration provides the ability to count cells within defined functional groups, although the investigator has to know what to count and cannot identify unknown diversity [27]. Developing metagenomic and metatranscriptomic techniques will contribute considerably to our understanding of the ecosystem in the coming years [28,29,30,31]. The first will provide access to the functional gene composition of microbial communities, while the second will allow for the identification of expressed transcripts in the microbiome [28]. Of note, another powerful ‘omic’ technology is metabolomics, through which it is possible to investigate the complex host–microbiota relationship via characterization of the microbial metabolism [32,33].

The current picture of the gut ecosystem is one of a very diverse, highly individual, yet functionally conserved ecosystem, and likewise the prebiotic concept has continued to evolve. According to the definition proposed in 2008 during the 6th meeting of the International Scientific Association of Probiotics and Prebiotics (ISAPP) in Ontario (Canada), a dietary prebiotic is ‘a selectively fermented ingredient that results in specific changes, in the composition and/or activity of the gastrointestinal microbiota, thus, conferring benefit(s) upon host health’ [34] and Table 1. Whilst similar to the original, this definition has some important differences. First, the reference to ‘one or a limited number’ has gone since it is apparent that prebiotics can bring about much more widespread changes than this. Second, the words ‘dietary’ and ‘gastrointestinal’ were inserted with the idea to extend the concept to other complex microbial ecosystems: such as a ‘skin prebiotic’, a ‘oral prebiotic’, or a ‘vaginal prebiotic’. In 2017, the ISAPP consensus panel proposed another definition of a prebiotic, i.e., ‘a substrate that is selectively utilized by host microorganisms conferring a health benefit’ [35]. The term substrate aligns with the word ‘utilized’ and implies ‘for growth through nourishment’, excluding viable microorganisms and antimicrobial agents as prebiotics [35]. The definition is more straightforward to avoid unnecessary technical jargon and, like the 2008 definition, clarifies that the targets extend beyond stimulation of bifidobacteria and lactobacilli while recognizing the health benefits that can derive from other beneficial taxa [35]. It also first discusses substrates that, although not defined as ‘prebiotic’, can affect composition of the microbiota through mechanisms not involving selective utilization by host microorganisms. Examples include minerals, vitamins, and bacteriophages.

Leaving definitions aside, the way that the concept has been interpreted by investigators has also evolved. In the early days, a rather simplistic differentiation into beneficial versus harmful bacteria prevailed, and this resulted in an over-emphasis on stimulating populations of bifidobacteria. They were one of the major cultivable groups and had known beneficial properties in the context of probiotics. Concomitantly, bacteroides were frequently dismissed as harmful, although in reality this is a huge and diverse group of bacteria with a range of impacts on the host, positive and negative [36,37,38,39].

With the two more recent ISAPP definitions [34,35], a more modern and nuanced understanding of prebiotics now fortunately has emerged. While the central concept of selective stimulation of part of the microbiota remains and is as valid today as it was in the 1990s, the understanding of what this means in terms of microbial population and activity has developed [11]. Bacteria with potential beneficial effects in IBD and obesity, such as the butyrate-producing *Faecalibacterium prausnitzii,* and the mucinophilic *Akkermansia muciniphila* respectively, have been identified, and more will certainly be identified as we increase our understanding of the ecosystem [40,41,42,43,44]. In fact, over recent years, there has been a shift in focus away from a simplistic differentiation between beneficial and harmful bacteria towards research aiming to understand ecological and functional features of the microbiota relevant for host physiology [9,45,46,47].

In addition, recent advances in understanding the wide functions that short chain fatty acids (SCFA) play in human physiology [48,49] has resulted in a focus on manipulation of metabolite profiles in the gut rather than simply changing bacterial populations. Metabolomics, in this case, represents an excellent tool to investigate the complex relationship between microbiota and host physiology. Thus, knowledge in this field is rapidly extending, suggesting encouraging new opportunities to improve human health. However, it is necessary to embrace all techniques available to investigate the gut microbiome, in a way that none is superior to the others, while still providing complementary information. Intelligent use of sequencing, quantitative PCR, FISH probing, and metabolomics can provide a true systems biology-based approach to study prebiotics and their impact on host health. With this approach, it is likely we will gain a much more sophisticated understanding of how prebiotics work, which will further allow the concept to evolve.

### 2.2. In Vitro Models of Colonic Fermentation

As part of an integrated approach to comprehensively study the functions of prebiotics on the gut microbiota, gut models, before moving, are particularly suited to in vivo animal testing or clinical studies in humans. In vitro gut models allow for study of the mechanistic effects of dietary, microbial, drug, and physiological factors on gut microbiota in a highly controlled environment and at levels that cannot be reached by an in vivo setup, and independent of the host. Therefore, in recent years, these models have evolved rapidly overcoming the issues associated with ethical concerns and providing with cost-effective tools for animal and human research.

**Figure 1:**
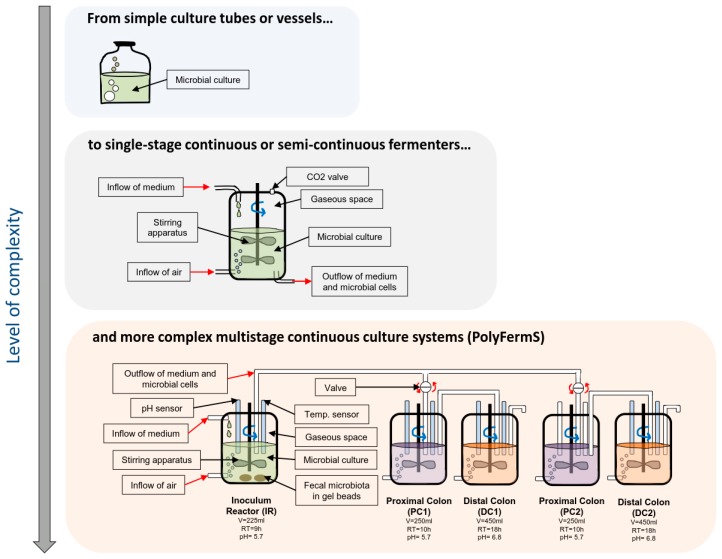
Schematic representation of the culture systems developed to analyze the microbiota ecosystems.

A range of systems has been developed over the past decades to model fermentation of the colon (see Figure 1), which harbors the highest density of microbes. This ranges from simple anaerobic batch culture systems in flasks to sophisticated multi-stage continuous flow models [52,53]. It should be emphasized that all models are different with respect to conditions and output. Therefore, the selection of the most suitable model should be done carefully, considering their features and limits in relation with the scientific question addressed. In particular, most models do not reproduce the sessile state bacterial populations in the colon and do not reach the high bacterial density and microbial competition of the gut [52]. A recent approach to solve these limitations has been developed as part of the PolyFermS system including a process of immobilization of fecal microbiota in gel beads mimicking cell density and competition of in vivo gut microbiota [54,55,56] (Figure 1). PolyFermS gut models can be expanded to various configurations including infant, elderly, or obese donors, allowing for a comparison of a control with different treatment effects with the same microbiota, ideal for investigating mechanisms of action and bacterial metabolite profiles of multiple prebiotics. For example, recent experiments confirmed that galacto-oligosaccharides (GOS), xylo-oligosaccharides (XOS), and beta-glucans are well metabolized by a healthy adult microbiota with marked shifts in the overall metabolism, and increased beneficial SCFA, such as butyrate and propionate. Although the use of different donors in these experiments do not allow linking these beneficial effects to specific phylogenetic group responses, donor-specific responses were obtained for three different microbiota representing two different enterotypes. These findings illustrate the sensitivity and value of data obtainable from in vitro gut models and emphasize the importance of considering that prebiotics may stimulate different bacterial groups and metabolic pathways according to the subject microbiota profile.

### 2.3. Microbiome-Modulating Compounds That May Impact Host Health

There are several compounds that appear to affect the composition of the gut microbiota via mechanisms that are apparently unrelated to a selective utilization by, or nourishment of, host microorganisms. A well-researched example includes the effect of vitamin B2 (riboflavin) and the growth of *Faecalibacterium prausnitzii.* The latter is a dominant beneficial bacterium, which is strictly anaerobic and accounts for 5–15% of the total number of bacteria in the human gut [57]. It is also one of the main butyrate producers in the gut with proven anti-inflammatory properties [42,43,58]. The number of *F. prausnitzii* is reduced in patients with inflammatory bowel disease, especially with Crohn’s disease [42,59,60,61] probably due to the increased oxidative stress created by the inflamed gut.

It has been shown that *F. prausnitzii* has a special ability to use riboflavin as an extracellular electron transporter that allows it to tolerate limited amounts of oxygen [62,63]. Although the oxygen tolerance provided by riboflavin is limited and does not facilitate the aerobic growth of this obligatory anaerobic bacteria, the stimulation of *F. prausnitzii* growth by the use of riboflavin might be a function of this vitamin and could be considered a microbiome-modulating property with potential clinical interest [62,63,64]. To test this hypothesis in humans, Harmsen and colleagues recently performed a pilot experiment in healthy male adults and found that riboflavin, indeed, increased the number of *F. prausnitzii* per gram of feces, and the number dropped again, although not to the baseline levels, after a one-week washout period [64]. This effect was paralleled by an increase in another group of anaerobes, *Roseburia* species, and a decrease in *Escherichia coli*, indicating an improvement in the anaerobic conditions and redox state in the gut [64]. Assuming that growth of other anaerobic beneficial microbes would be possible under low aerobic conditions using vitamins, this will open up new possibilities both in the fabrication and in the application of such probiotic–vitamin combinations as supplements for prevention of human diseases, using such microorganisms that otherwise would not survive the normal (i.e., oxygenated) atmosphere.

Beside vitamin B2, other non-carbohydrate compounds can modulate the gut microbiota either through ‘selective utilization’ or other mechanisms and exert beneficial effects on the host. The polyphenols contained in red wine, for example, were demonstrated to modulate the growth of selected gut microbiota (increased growth of *Bacteroides* and *Bifidobacterium* genera) in humans [65]. The changes in gut microbiota following red wine consumption were associated with a reduction in triglycerides and cholesterol, linking polyphenol intake to cardiovascular benefits for the host [65] and confirmed by a reduction in the concentration of the C-reactive protein inflammatory marker. A microbiome-modulating effect may also exist with minerals such as calcium [66] and iron [67,68], and a combination of prebiotics and micronutrients [56,69]. Mice fed a high fat diet supplemented with calcium showed lower plasma levels of the endotoxin LPS and a leaner phenotype, and this was associated with the growth of *Bacteroides* and *Bifidobacterium* [66]. Finally, very recent studies also show that supplementation with omega-3 polyunsaturated fatty acids (PUFA) may modify the human gut microbiota [70,71]. Specifically, Menni and collaborators showed that, in a population consisting of middle-aged and elderly women, circulating levels of omega-3 fatty acids are associated with higher microbiome diversity and with a higher abundance of bacteria belonging to the family of the short chain fatty acid-producer *Lachnospiraceae* [70]. Similarly, Watson and collaborators observed a reversible increased abundance of several genera, including *Bifidobacterium, Roseburia*, and *Lactobacillus* following omega-3 PUFA supplementation [71].

Taken all together, these data suggest that several non-carbohydrate compounds modulate the gut microbiota, but whether all of them qualify as prebiotics is still under debate.

## 3. Discussion and Conclusions

Although the prebiotic definition has evolved together with the technological developments that have characterized the last twenty years of microbiology research, what exactly qualifies as a prebiotic continues to be a matter of debate. According to the most recent ISAPP definition [35], a prebiotic is ‘a substrate that is selectively utilized by host microorganisms conferring a health benefit’. While the central concept of selective stimulation of part of the microbiota remains valid, the understanding of what this means in terms of microbial population and activity has developed. Bifidobacteria and lactobacilli do not represent the only functionally important members of gut microbiota, and metabolic cross-feeding phenomena enhance the complexity of the gut bacterial ecosystems. The true challenge is, thus, to relate a prebiotic to specific ecosystem shifts and/or metabolite production and finally to the health benefits for the host.

Sophisticated in vitro models mimicking colonic fermentation to study the effects of prebiotics on indicator species and overall composition, as well as metabolic products, have recently been developed. However, they also exhibit limitations. For example, they only simulate the capacity of the microbiome to ferment/utilize a certain prebiotic but do not indicate the functional implications associated with those changes. While butyrate enhancement can be assessed in vitro, this does not always predict the true interaction of the gut microbiota with the host cells or host-derived soluble factors [72]. For that, additional data is needed that should be generated by integrating data from in vitro and in vivo systems with human studies. An addition to these systems is represented by the intestinal organoids: three-dimensional in vitro tissue models that retain many of the physiologically relevant features of the in vivo intestinal tissue [73,74]. One important feature of these organoids is that they are robust models in the presence of bacterial and viral challenges allowing for true co-culture experiments in which epithelia and microbes can be maintained for extended periods of time in the same culture dish [73]. Therefore, they represent a modular and highly adaptable model system for evaluating the molecular basis of the host–microbe interface [73].

Scientists will continue to evolve the concept of prebiotics based on the increasing understanding of the effects of various nutritional compounds on microbiota composition and metabolite production. This includes the most recent findings of non-carbohydrate compounds such as polyphenols, minerals, nutritional lipids, and vitamins, which so far have not been considered for their potential effects on gut microbiota. This is not surprising considering that, under physiological conditions, at least in the case of vitamins and lipids, most of them are absorbed in the upper small intestine but do not reach distal parts of the GI tract. Nevertheless, whether their effects on host physiology are mediated, at least in part, via modulating the gut microbiome is unknown and warrants further investigation.

## Figures and Tables

**Table 1:** Main prebiotic definitions evolved from 1995 until 2017.

Year	Prebiotic Definition	Reference
1995	A prebiotic is a nondigestible food ingredient that beneficially affects the host by selectively stimulating the growth and/or activity of one or a limited number of bacteria in the colon, and thus improves host health.	[19]
2003	Prebiotics are nondigestible substances that provide a beneficial physiological effect on the host by selectively stimulating the favorable growth or activity of a limited number of indigenous bacteria.	[50]
2004	A prebiotic is a selectively fermented ingredient that allows specific changes, both in the composition and/or activity in the gastrointestinal microflora that confers benefits upon host wellbeing and health.	[51]
2010	A prebiotic is a selectively fermented ingredient that results in specific changes in the composition and/or activity of the gastrointestinal microbiota, thus conferring benefit(s) upon host health.	[34]
2017	A prebiotic is a substrate that is selectively utilized by host microorganisms conferring health benefit.	[35]
